# The Role of Epiretinal Membrane on Treatment of Neovascular Age-Related Macular Degeneration with Intravitreal Bevacizumab

**DOI:** 10.1155/2013/958724

**Published:** 2013-12-24

**Authors:** Zeynep Alkin, Abdullah Ozkaya, Ozen Ayranci Osmanbasoglu, Alper Agca, Yalcin Karakucuk, Ahmet Taylan Yazici, Ahmet Demirok

**Affiliations:** ^1^Beyoglu Eye Training and Research Hospital, Bereketzade Cami Sok No. 2 Beyoglu, Istanbul, Turkey; ^2^Medeniyet University, Goztepe, 34700 Istanbul, Turkey

## Abstract

*Purpose*. To determine the effect of epiretinal membranes (ERM) on the treatment response and the number of intravitreal bevacizumab injections (IVB) in patients with neovascular age-related macular degeneration (nAMD). *Methods*. A retrospective chart review was performed on 63 eyes of 63 patients. The patients were divided into AMD group (*n* = 35) and AMD/ERM group (*n* = 28). Best corrected visual acuity (BCVA) and central retinal thickness (CRT), as well as the number of injections, were evaluated. *Results*. There was a significant improvement in BCVA at 3 months for the AMD and AMD/ERM groups (*P* = 0.02, *P* = 0.03, resp.). At 6, 12, and 18 months, BCVA did not change significantly in either of the groups compared to baseline (*P* > 0.05 for all). At 3, 6, 12, and 24 months, the AMD group had an improvement in BCVA (logMAR) of 0.09, 0.06, 0.06, and 0.03 versus 0.08, 0.07, 0.05, and 0.03 for the AMD/ERM group (*P* = 0.29, *P* = 0.88, *P* = 0.74, *P* = 0.85, resp.). A significant decrease in CRT occurred in both groups for all time points (*P* < 0.001 for all). The change in CRT was not statistically different between the two groups at all time points (*P* > 0.05 for all). The mean number of injections over 24 months was 8.8 in the AMD group and 9.2 in the AMD/ERM group (*P* = 0.76). *Conclusion*. During 24 months, visual and anatomical outcomes of IVB in nAMD patients were comparable with those in nAMD patients with ERM with similar injection numbers.

## 1. Introduction 

Neovascular age-related macular degeneration (nAMD) is the leading cause of vision loss worldwide among people aged 50 years and older [1–3].

Vascular endothelial growth factor (VEGF) plays a major role in choroidal neovascularization (CNV) secondary to nAMD characterized by angiogenesis and increased vascular permeability. This condition leads to abnormal fluid collection within or below the retina [4–6]. Since the first introduction of the off-label use of intravitreal bevacizumab (IVB) for neovascular AMD in 2005, numerous studies have reported the efficacy and safety of this treatment [7]. Regarding ocular and systemic safety issues, as well as cost and patient convenience concerns, clinical trials with flexible dosing regimens have been released to allow for fewer injections of bevacizumab and ranibizumab [8–10]. The PrONTO study, which evaluated an optical coherence tomography (OCT)-based retreatment regimen for ranibizumab in nAMD, showed that it is possible to stabilize and improve visual acuity with an as-needed dosing regimen while reducing the number of injections over 24 months [11]. Similarly, ABC trial demonstrated that IVB was superior to standard care when administered on an as-needed treatment regimen for every six weeks after the initial loading phase of three injections [12].

Idiopathic epiretinal membranes (ERMs) cause macular structural changes such as retinal folds, vascular leakage, macular thickening, cystoid macular edema, pseudohole formation, foveal ectopia, and foveal detachment by tractional forces on the retinal surface [13]. Both idiopathic ERMs and nAMD are observed in the same age group and cause visual acuity deterioration [14]. In clinical practice, the coexistence of the aforementioned diseases in advanced age groups may cause difficulties in the management of AMD. The study by Pierro et al. identified the coexistence of an ERM in 26% of eyes with nAMD using an SD-OCT system [15]. However, no study has investigated whether ERM could adversely affect the course of nAMD or change the treatment strategy.

In this retrospective study, we performed a comparative assessment to determine whether the effects of IVB were different between the nAMD patients with ERM and without ERM on treatment response.

## 2. Methods

This is a retrospective study which included the patients who had undergone IVB treatment for newly diagnosed treatment naïve nAMD at Beyoglu Eye Training and Research Hospital between July 2007 and January 2009. Approval for data collection and analysis was obtained from the ethics committee of the hospital, and all patients provided informed consent. The methodology of the study was conducted in accordance with the tenets of the Helsinki Declaration.

The inclusion criteria for the study were as follows: (1) age of 50 years or more; (2) the presence of active leakage pattern indicating subfoveal or juxtafoveal CNV on FA; (3) all subgroups of CNV including predominantly classic or minimal classic/occult; (4) baseline best corrected visual acuity (BCVA) of ≥20/400. Patients with coexisting diseases involving the posterior pole such as diabetic retinopathy, vascular occlusions, inflammatory diseases, trauma, high myopia, vitreomacular interface diseases other than ERM, and cataract development or progression during the follow-up were excluded from the analysis. Patients who had history of ocular surgery within the last 6 months or had previously undergone vitreoretinal surgery, intravitreal injection, or photodynamic therapy were also excluded.

All patients underwent a complete ophthalmologic examination, which included BCVA testing with standardized refraction using early treatment diabetic retinopathy study (ETDRS) charts, a fundus examination with a 90-diopter indirect lens after mydriasis, colour fundus photography, and FA.

All patients received a loading dose of three consecutive injections of bevacizumab (1.25 mg/0.05 mL) with 4 to 6 weeks intervals in the initial phase. Patients were then followed up by 4 to 6 weeks intervals with clinical examinations and with OCT. After three initial loading doses, the indication for intravitreal reinjections was based on VA loss (five ETDRS letters or more), an increase in central retinal thickness (CRT) of at least 50 *μ*m from the lowest recorded value, new macular haemorrhage, or leakage on FA. In the eyes with ERM, if the persistence of intraretinal fluid after re-injection of bevacizumab was seen, no additional injection was performed until progressing edema occurred. Persistent serous retinal pigment epithelial detachment (PED) was not a retreatment criterion.

At follow-up visits, FA was repeated only when the cause of VA deterioration could not be clarified with the clinical examination and OCT. For OCT imaging, time-domain OCT was performed (Stratus OCT 3000, Carl Zeiss, Meditec Inc., Dublin, CA, USA) after mydriasis. CRT, defined as the mean thickness of the neurosensory retina in a central 1 mm diameter area, was computed using OCT mapping software generated by the device.

Patients were divided into two groups based on the presence or absence of accompanying ERM: AMD group and AMD/ERM group. In the AMD/ERM group, in addition to nAMD, ERM covering the central macular area which was grade 3 (retinal vascular distortion and/or retinal folds) based on the ophthalmoscopic examination was present [16]. The diagnosis of ERM was then confirmed by the presence of a hyperreflective band indicating a preretinal membrane adherent to the inner retinal surface, as observed on OCT.

All injections were performed under sterile conditions after topical anesthesia and 10% povidone-iodine (Betadine; Purdue Pharma, Stamford, CT) scrub was used on the lids and lashes, and then 5% povidone-iodine was administered on the conjunctival sac. IVB was injected through the pars plana at 3.5 mm to 4 mm to the limbus with a 27-gauge needle. Patients were then instructed to consult the hospital if they experience decreased vision, eye pain, or any new symptoms.

Data collected from the patients' records included age, gender, type of CNV (predominantly classic or minimal classic/occult), the presence of PED at baseline, and BCVA and CRT at baseline and at the 3-, 6-, 12-, and 24-month follow-up visits. Also, injection numbers at 24-month follow-up were recorded.

The primary outcome measures included the changes in BCVA and CRT between baseline and 3-, 6-, 12-, and 24-month follow-up. Secondary outcome measure was the number of injections at 24-month follow-up.

### 2.1. Statistical Analysis

Visual acuity was converted to logarithm of minimum angle of resolution (logMAR) for statistical analysis. The mean changes in BCVA and CRT over time were analyzed using one-way ANOVA with repeated measures. Fisher's exact test was used to compare nominal parameters between the groups, and Mann-Whitney *U* test was used for continuous parameters. The statistical analysis was performed using SPSS version (Version 15.0, SPSS Inc., Chicago, IL, USA). A *P* value of less than 0.05 was considered to be statistically significant.

## 3. Results

A total of 63 eyes of 63 patients were included in this study. There were 35 patients (15 female and 20 male) in the AMD group and 28 patients (11 female and 17 male) in the AMD/ERM group. The mean age was 66.3 ± 9.9 years in the AMD group and 68.6 ± 9.4 years in the AMD/ERM group. The baseline information of the groups, including the demographic data and ocular characteristics, is listed in Table 1. No baseline parameter significantly differed between the groups.

**Figure 1:**
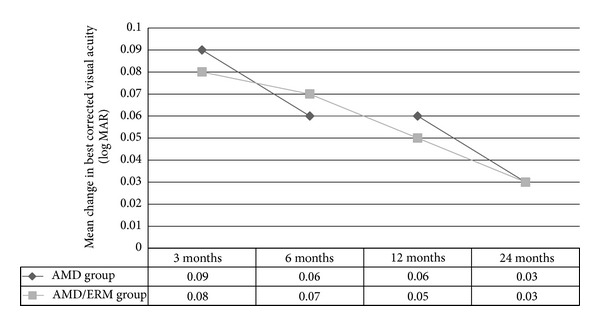
Change in best corrected visual acuity (logMAR) from baseline and to the 3-, 6-, 12-, and 24-month follow-up in the AMD group and AMD/ERM group. (Respective intravitreal injections administered at months 0, 1, and 2 and then as per treatment protocol.)

Mean BCVA values at baseline and at the 3-, 6-, 12-, and 24-month follow-up were listed in Table 2. There was no significant difference in the BCVA improvement between the groups at the 3-, 6-, 12-, and 24-month follow-up (*P* = 0.29, *P* = 0.88, *P* = 0.74, *P* = 0.85, resp.) (Figure 1).

In the AMD group, BCVA did not change (±5 ETDRS letters) in 22 eyes (63%), improved >5 ETDRS letters in 9 eyes (26%), and decreased >5 ETDRS letters in 4 eyes (11%) at 24 months. In the AMD/ERM group, BCVA did not change (±5 ETDRS letters) in 16 eyes (57%), improved >5 letters in 7 eyes (25%), and decreased >5 ETDRS letters in 5 eyes (18%) at 24 months. Distribution of changes in BCVA (maintenance, improvement, or decrease of BCVA) was not statistically significant between the groups (*P* = 0.65, *P* = 0.94, *P* = 0.47, resp.).

**Figure 2:**
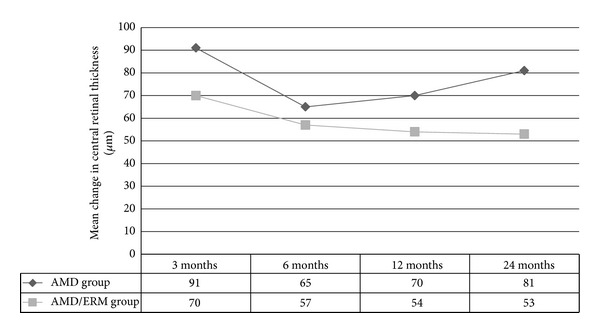
Change in central retinal thickness (*μ*m) from baseline and to the 3-, 6-, 12-, and 24-month follow-up in the AMD group and AMD/ERM group. (Respective intravitreal injections administered at months 0, 1, and 2 and then as per treatment protocol.)

Mean CRT values at baseline and at the 3-, 6-, 12-, and 24-month follow-up were listed in Table 2. The mean change in CRT was not statistically different between the groups at the 3-, 6-, 12-, and 24-month follow-up (*P* = 0.13, *P* = 0.52, *P* = 0.94, *P* = 0.17, resp.) (Figure 2).

The mean number of injections performed per eye during 24-month follow-up was 8.8 (range from 3 to 15) for the AMD group and 9.2 (range from 3 to 16) for the AMD/ERM group. This difference was not statistically different between the groups (*P* = 0.76). During the following 21 months after three initial loading doses, 3 patients (8%) in the AMD group and only one patient (3%) in the AMD/ERM group did not require further treatment. During the 24-month follow-up, no serious injection- or drug-related ocular or systemic adverse events were observed.

## 4. Discussion

In this study, we compared the visual and anatomical outcomes and frequency of injections of an as-needed treatment regimen following a loading dose of three initial injections with bevacizumab between patients with nAMD without an ERM and those with an ERM. By 24 months, similar improvements in both groups for BCVA occurred after the initial loading dose and then showed a gradual decrease through the study. The comparison of AMD treatment trials (CATT) was the first trial to provide evidence supporting the use of bevacizumab therapy in nAMD [17]. In the CATT, patients were randomly assigned to receive intravitreal ranibizumab or bevacizumab on a monthly schedule or on an as-needed treatment regimen which was largely driven by fluid on OCT. The CATT 2-year report showed that as-needed bevacizumab group gained 5 ETDRS letters (equal to 0.1 logMAR) compared to baseline. In our study, both AMD and AMD/ERM groups achieved lower gains in BCVA at 24 months than CATT. In the CATT, mean baseline letter score was 60 ETDRS letters (equal to 0.5 logMAR) which is better than that of the present study with the values of 0.7 logMAR for AMD group and 0.73 logMAR for AMD/ERM group. In our study, worse baseline visual acuity levels may be responsible for lower VA gain than CATT.

A significant reduction in the baseline CRT values in both groups reflected the efficacy of IVB even in the presence of ERM. The reduction in CRT after an anti-VEGF injection is primarily the result of the reduced permeability of the neovascular lesion, with a subsequent reduction in intra- and subretinal fluid levels [18]. In our study, the two groups differed in mean CRT at baseline, but this difference was not statistically significant. Previous studies revealed that patients with macular edema secondary to epiretinal membranes had both cystic and noncystic changes on OCT images that result in increased CRT values [19]. In addition, Chen et al. found that intravitreal bevacizumab injection had no beneficial effect on CRT improvement for eyes with persistent macular edema after idiopathic macular ERM removal [20]. Thus, we suggest that the higher consistency of CRT values in the AMD-ERM group than in the AMD group throughout the study period likely resulted from ERM-related macular edema.

Different doses and treatment protocols have been utilized in the management of CNV with bevacizumab. Most regimens involve an as-needed or PRN regimen. This may include one to three loading doses which was followed by an as-needed treatment phase based on OCT and FA changes [8–10]. As such, the best criteria for retreatment are uncertain, particularly in the presence of an ERM. When considering retreatment in nAMD, FA has been shown to be unable to differentiate between leakage and staining, and poor agreement persists in the interpretation of FA in nAMD among physicians [21, 22]. We also commented on the difficulty of correctly identifying leakage due to CNV in the presence of ERM, which can confound the interpretation of FA images. Fluid can accumulate in the subretinal or sub-RPE spaces or between all layers of the inner retina and can be quantitatively evaluated with OCT. It has been suggested that subretinal fluid may be a more sensitive and clinically relevant parameter to guide treatment regimens than CRT. In a study by Golbaz et al., a manual segmentation analysis using SD-OCT of the intra- and subretinal compartments in nAMD revealed an immediate and accentuated response in subretinal fluid values during anti-VEGF therapy [23]. Furthermore, as noted in previous studies, larger CNV area and presence of GA were independently associated with less improvement in VA [24]. It is important to note that the discrimination of intra- or subretinal fluid and such variables between the groups could not be employed in this study because of its retrospective design. We are aware that this would affect our results.

After the loading phase, the eyes responding minimally or with no anatomic or functional improvement to two consecutive injections of bevacizumab in the AMD/ERM group did not receive further therapy until the retreatment criteria were met. Because further injections may not be of benefit in this case, we assumed that unnecessary reinjections could be eliminated by this way in the presence of an ERM. In this study, both groups required a similar number of injections on as-needed treatment regimen during the study period. These results suggest that a retreatment strategy based on OCT can be implemented with anti-VEGF agents when making retreatment decisions even in the presence of ERM.

Several published studies have shown a higher rate of abnormalities of the vitreomacular interface, including ERMs, retinal thickening, and retinal distortion, in patients with AMD compared to those without AMD [25–27]. Pierro et al. showed that an ERM was present in 26% to 32% of eyes with nAMD [15]. It has been speculated that chronic traction on the retina may cause the degeneration or alteration of the retinal pigment epithelium or Bruch membrane. Mechanical traction may create low-grade inflammation and stimulate the induction or progression of nAMD by inducing the continued release of VEGF [28].

According to the theory for the role of mechanical factors in ERMs local forces induce a release of mediators that lead to a breakdown of the blood-retinal barrier, resulting in macular edema [13]. Furthermore, these tractional forces may antagonize the effects of anti-VEGF treatments and cause pharmacological resistance in those patients [29, 30]. Unfortunately, no published study has investigated whether there is an increase in vitreous VEGF levels in the presence of vitreoretinal interface pathologies or an alteration in the penetration of anti-VEGF drugs through the ERM in eyes with nAMD. Luttrull and Spink showed that vitrectomy with ERM peeling may provide visual improvement in selected patients with nAMD after initiation of anti-VEGF therapy in a small, uncontrolled, nonrandomized, and retrospective study [31]. Similarly, Mojana et al. found that vitrectomy may be beneficial to improve vision in some of the patients with vitreomacular traction who do not respond to anti-VEGF therapy for nAMD [30]. However, this series had a small number of patients to conclude. In addition, vitrectomy in such eyes can result in complications and may also complicate anti-VEGF treatment, as it may shortens the half-life of intravitreal anti-VEGF drugs, so that higher doses and more frequent injections of anti-VEGF drugs may be needed postoperatively [32, 33]. Based on these findings, none of the patients in our study had vitrectomy for ERM peeling during the study period.

The results of this study should be interpreted with consideration of the retrospective data collection and the small number of patients. Further studies will be helpful for understanding how ERM and vitreoretinal surface disorders affect the dosing strategy of anti-VEGF agents and the course of nAMD in the presence of ERM.

In summary, the presence of an ERM can be an additional cause of macular edema in nAMD patients and should also be considered in clinical practice. While the quantitative measurement of retinal thickness by OCT has been used as a retreatment criterion in neovascular AMD therapies, the qualitative evaluation and detection of vitreoretinal interface abnormalities have been important in decisions regarding treatment with intravitreal antiangiogenic agents.

## Figures and Tables

**Table 1:** Comparison of patient demographics and characteristics of the AMD and AMD/ERM groups at baseline.

	AMD group	AMD/ERM group	*P* value
Number of patients	35	28	—
Mean age, years ± SD (range)*	66.3 ± 9.9 (52–84)	68.6 ± 9.4 (55–85)	0.38
Gender (female/male)^†^	15/20	11/17	0.77
CNV type classic/nonclassic^†^	12/23	11/17	0.68
Mean BCVA, logMAR ± SD (range)*	0.71 ± 0.28 (0.3–1.3)	0.73 ± 0.31 (0.3–1.3)	0.88
Mean CRT, *μ*m ± SD (range)*	315 ± 102 (275–462)	371 ± 95 (298–519)	0.06
PED (%)^†^	16 (45%)	9 (32%)	0.27

AMD, age-related macular degeneration; ERM, epiretinal membrane; BCVA, best corrected visual acuity; CRT, central retinal thickness; PED, pigment epithelium detachment; SD, standard deviation.

*Mann-Whitney *U* test.

^†^Chi-square test.

**Table 2:** Mean best corrected visual acuity and central retinal thickness values at baseline and at the 3-, 6-, 12-, and 24-month follow-up for AMD and AMD/ERM groups.

	AMD group	*P* value	AMD/ERM group	*P* value
Mean baseline BCVA, logMAR ± SD (range)	0.71 ± 0.28 (0.3–1.3)		0.73 ± 0.31 (0.3–1.3)	
Mean BCVA at 3 months, logMAR ± SD (range)	0.61 ± 0.22 (0.2–1.0)	0.02	0.64 ± 0.32 (0.3–1.1)	0.03
Mean BCVA at 6 months, logMAR ± SD (range)	0.63 ± 0.21 (0.3–1.0)	0.11	0.66 ± 0.31 (0.3–1.1)	0.13
Mean BCVA at 12 months, logMAR ± SD (range)	0.63 ± 0.22 (0.3–1.0)	0.13	0.67 ± 0.28 (0.3–1.2)	0.21
Mean BCVA at 24 months, logMAR ± SD (range)	0.66 ± 0.24 (0.3–1.0)	0.39	0.70 ± 0.25 (0.3–1.3)	0.52
Mean baseline CRT, *μ*m ± SD (range)	315 ± 102 (275–462)		371 ± 95 (298–519)	
Mean CRT at 3 months, *μ*m ± SD (range)	224 ± 63 (172–284)	<0.001	301 ± 86 (221–353)	<0.001
Mean CRT at 6 months, *μ*m ± SD (range)	257 ± 71 (178–299)	<0.001	322 ± 90 (263–367)	<0.001
Mean CRT at 12 months, *μ*m ± SD (range)	245 ± 73 (160–312)	<0.001	311 ± 67 (232–380)	<0.001
Mean CRT at 24 months, *μ*m ± SD (range)	225 ± 62 (153–302)	<0.001	314 ± 77 (240–391)	<0.001

AMD, age-related macular degeneration; ERM, epiretinal membrane; BCVA, best corrected visual acuity; CRT, central retinal thickness; SD, standard deviation.

*One-way ANOVA test for all comparisons.
